# Early response prediction during radiotherapy in rectal cancer using sequential diffusion-weighted imaging at a magnetic resonance image-guided linear accelerator

**DOI:** 10.1016/j.phro.2025.100846

**Published:** 2025-10-08

**Authors:** Johanna A. Hundvin, Jonas Habrich, Cihan Gani, Jörg Assmus, Inger Marie Løes, Sara Pilskog, Kathrine R. Redalen, Daniela Thorwarth

**Affiliations:** a Cancer Clinic, Haukeland University Hospital, Bergen, Norway; b Institute of Physics and Technology, University of Bergen, Bergen, Norway; c Section for Biomedical Physics, Department of Radiation Oncology, University Hospital Tübingen, Tübingen, Germany; d Department of Radiation Oncology, University Hospital Tübingen, Tübingen, Germany; e Centre for Clinical Research, Haukeland University Hospital, Bergen, Norway; f Department of Physics, Norwegian University of Science and Technology, Trondheim, Norway

**Keywords:** Biomarker, MRI, Diffusion MRI, Apparent diffusion coefficient, ADC

## Abstract

**Background and purpose:**

Varying response to chemoradiotherapy (CRT) challenges the treatment of locally advanced rectal cancer (LARC). Our purpose was to timely stratify responders by investigating the predictive potential of sequential, in-treatment diffusion-weighted (DW) magnetic resonance (MR) images.

**Materials and methods:**

DW images were acquired with a 1.5 T MR-Linac at baseline and during MR-guided CRT in 15 patients with LARC prescribed long-course CRT, prospectively enrolled between 2018 and 2021. Tumour response was classified as good or poor based on pathologic tumour regression. Changes in mean tumour apparent diffusion coefficient (ADC) were analysed by linear mixed-effects models and compared to modelling using volume. Model coefficient analysis and receiver operating characteristics (ROCs) with area under the curve (AUC) were applied for timepoint investigation of response.

**Results:**

Six patients had good response, with significant difference in median (range) baseline ADC; ADC_good_ = 1.3 (1.1–1.5) ∙ 10^-3^ mm^2^/s, ADC_poor_ = 1.1 (0.9–1.4) ∙ 10^-3^ mm^2^/s, (p = 0.03), and with greater ADC change at all evaluated timepoints. This resulted in AUCs of 0.73–0.85 and the steepest slope (m) after five fractions (m_good_ = 0.090, m_poor_ = 0.014). Combining baseline and slope improved the differentiation with AUCs of 0.90, 0.87, 0.85 and 0.83 after 5, 10, 13 or all fractions, respectively. Stratification based on volume changes was feasible after 15/25 fractions.

**Conclusions:**

Early indication of treatment response in LARC was achieved by combining baseline and sequential ADC information. These encouraging results should be validated in a larger cohort.

## Highlights

• Diffusion images from MR-guided radiotherapy monitor rectal cancer treatment response.
• Response to radiotherapy was predicted as early as first treatment week.
• Combining baseline and sequential information enhance the response differentiation.

## Graphical abstract

**Figure:**
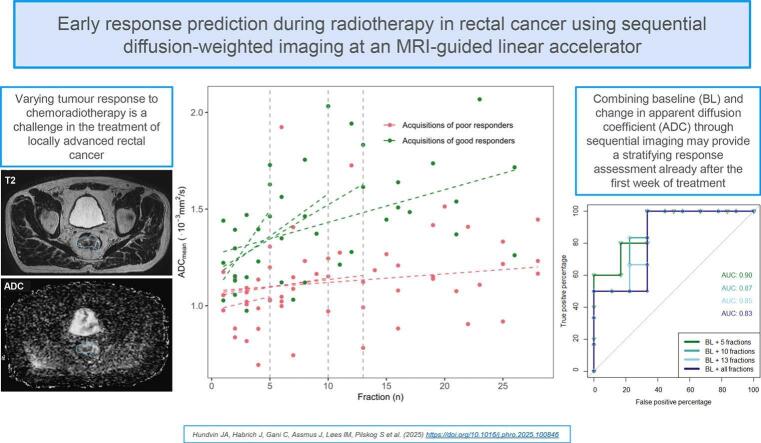

## 1 Introduction

Treatment of locally advanced rectal cancer (LARC) has classically consisted of preoperative chemoradiotherapy (CRT) followed by surgery irrespective of response and postoperative chemotherapy [1]. Over the last decade non-operative management in case of a clinical complete response has emerged as a treatment alternative with superior organ function compared with surgery [2,3]. However, a major obstacle is the varying tumour response and lack of early response prediction to timely adjust the treatment, as response evaluation is generally carried out after completion of CRT [[4], [5], [6]]. In cases of inadequate tumour response, early biomarkers predicting response could guide the personalised treatment choices aimed to achieve clinical or pathological complete tumour response (pCR) by intensifying with consolidation chemotherapy or radiotherapy dose escalation [7], or for the poorly responding patients to proceed directly to surgery without further delay or CRT side effects [8].

Magnetic resonance imaging (MRI) has a central role in both the initial staging and response evaluation after CRT of rectal cancer, with the capacity to stratify patients into different prognostic groups [9,10]. The apparent diffusion coefficient (ADC) is an MRI-based non-invasive biomarker, derived from diffusion weighted (DW)-MRI. While DW imaging is performed routinely to aid qualitative investigation of rectal cancer and local lymph nodes, the potential of the quantitative ADC parameter is still not fully understood [[11], [12], [13]]. The definition of ADC-threshold values for response prediction based on pre-CRT measurements is challenged by inconsistent study results [14,15]. A greater increase in ADC for the favourable compared to the unfavourable response group from CRT has been reported by many studies using images of the later part or end of radiotherapy course [14,15]. However, cellular damage and tumour oedema as a consequence of CRT has been shown to appear within days of initiating therapy, which encourage earlier timepoints for response assessment [16]. Previous studies focused on early timepoints for response assessment have shown inconclusive results, potentially influenced by the coarse image sampling of the subtle changes [[17], [18], [19], [20], [21], [22]]. No quantified level of ADC change nor ideal timepoint for early response evaluation have thus been established.

The recent introduction of integrated MRI and linear accelerator systems has enabled more frequent and non-invasive monitoring of biological changes during treatment. Systems operating at 1.5 T have demonstrated the capability to deliver high-precision radiotherapy [23], while providing sufficient image quality for accurate DW imaging [24]. The practical challenges of exploring varying timepoints for response assessment is thereby substantially reduced, with potential of providing a stronger ground for future ADC threshold analysis. The purpose of this study was hence to investigate the potential of sequential DW-MRI acquisitions in predicting response to CRT in patients with LARC treated using such MRI-guided radiotherapy platforms.

## 2 Materials and methods

All patients studied in the present report had LARC with an indication for long-course CRT according to national guidelines. Treatment was carried out at the 1.5 T MRI-guided linear accelerator (MR-Linac, Unity, Elekta AB, Stockholm, Sweden). All patients provided written informed consent for a prospective basket trial evaluating the feasibility of online adaptive MR-guided radiotherapy that covered the collection and evaluation of imaging data acquired on the 1.5 T MR-Linac. Between October 2018 and June 2021, a total of 22 patients were enrolled and treated on the MR-Linac. However, six patients had to be excluded from analysis upfront due to too small tumour volume for accurate delineation (3), prothesis (1), missing acquisitions (1), or reirradiation (1). One patient had a tumour too small to be reassessed during CRT and was only included in the baseline analysis. All patients received 50.4 Gy in 28 fractions to the primary tumour and regional lymph nodes by image-guided intensity modulated radiation therapy (IMRT) with concomitant 5-fluorouracil chemotherapy. No patient had distant metastases or mucinous tumour, while one patient received a boost (2.2 Gy/fr) to a lateral pelvic lymph node.

The study was approved by the local ethics committee (approval no. 659/2017B01, NCT04172753), and all patients provided written informed consent. Following surgery approximately 8–12 weeks after CRT, the tumour response was classified as good (Dworak 3 or 4) or poor (Dworak 0–2) based on pathologic tumour regression [25].

### 2.1 Image acquisition and ADC calculation

All patients were scanned in head-first supine position with knee fixation (KneeSTEP M, IT-V, Innsbruck, Austria). In addition to the anatomical T2-weighted MRIs required for patient positioning and treatment adaptation at the 1.5 T MR-Linac, an extended imaging protocol consisting of DW-, T1- and T2-weighted MRIs was acquired approximately twice weekly, using the radiolucent whole body receive coils of the MR-Linac. The DW-MRI scans were acquired using b-values of 0, 200, 500 and 800 s/mm^2^ (EPI4b). Starting in April 2021, the DW-MRI protocol was adapted according to the recommendations of the MR-Linac consortium and b-values of 0, 150 and 500 s/mm^2^ (EPI3b) were acquired [26]. Further details about the sequence parameters of both DW-MRI sequences are included in the Supplementary Material (Supplementary Table S1). The volume of interest (VOI) was defined as the primary gross tumour volume (GTV) and segmented axially on the highest b-value image with the corresponding T2-weighted image as visual reference, where the DW image took precedence in case of discordance. An in-house written python script was used for voxel-wise calculation of the ADC based on the mono-exponential model using b-values 150/200 and 500 s/mm^2^ [27]. The mean ADC value over the total GTV (ADC_mean_) was extracted using the open-source software 3D Slicer.

### 2.2 Statistical analysis

Descriptive methods were used to characterise the patients in the dataset. Normality of distributions was assessed through quantile–quantile (qq) plots and statistical differences tested by two-tailed t-tests. The differences between responders and non-responders over time was investigated estimating the longitudinal linear mixed-effects model of ADC depending on fraction (representing time), response and their interaction with an individual random intercept.

$${\mathrm{ADC}}_{\mathrm{mean}}=\beta_{0}+\beta_{1}\;∙\;fraction+\beta_{2}\;∙\;response+\beta_{3}\;∙\;fraction\;∙\;response+r\left(patient\right)$$

The interaction term in this model (β_3_) describes the change of the difference in ADC between the response groups from baseline. Estimation of β_3_ for an increasing number of fractions included in the model enabled us to observe how many fractions are needed until the estimate stabilises both in size and precision (95 % confidence intervals), that is, giving an estimate of how many fractions may be needed to distinguish between responders and non-responders. This model is robust for missing values assuming missing at random.

Though this study intended to investigate the value of sequential ADC imaging during RT, the same analysis was performed for volume, as previous studies indicate a possible predictive value of volumetric assessment after CRT [14]. As an additional sub analysis, the Early Regression Index (ERI) was calculated to retrospectively validate this promising biomarker utilised in the THUNDER-2 trial on our cohort [28]. Due to lacking acquisitions from fraction 12 for most patients (Supplementary Table S2) required for the ERI calculation (biological effective dose (BED) = 25.5 Gy), the ERI was calculated using baseline and the 12th or first following fraction (ranging from 13 to 16).

The area under the curve (AUC) of receiver operating characteristic (ROC) analysis was determined to quantitatively compare the models utilising different variations of initial and sequential tumour ADC_mean_. The expected stepwise ROC curve was less robust to variation than the models, as the ROC analysis inevitably limited the dataset to one datapoint per patient. In all cases, the level of significance was set to p < 0.05, where we abstained from testing significance between AUCs due to lack of power. All data were analysed using R (version 2024.09.0).

## 3 Results

**Fig. 1:**
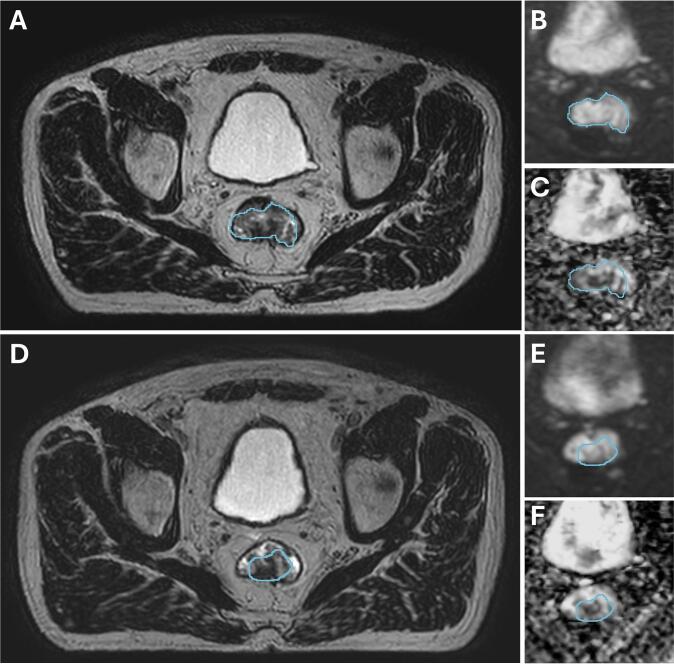
Illustrating images from patient 1, acquired at baseline (A, B, C) and the 5th fraction (D, E, F). The T2-weighted images (A, D) are shown with cropped sections of their respective b500 images (B, E) and apparent diffusion coefficient (ADC) maps (C, F). The GTV is delineated in blue. (For interpretation of the references to colour in this figure legend, the reader is referred to the web version of this article.)

There was no prominent difference in baseline patient characteristics between the two response groups (Supplementary Table S2). For the longitudinal analysis, all patients had an average of 10 acquisitions (range 6–17) over the course of RT, in addition to the baseline scan (Fig. 1, Supplementary Table S2). On an individual level (Supplementary Fig. S1), change in ADC_mean_ was varying while a decrease in tumour volume was seen irrespective of response group.

**Fig. 2:**
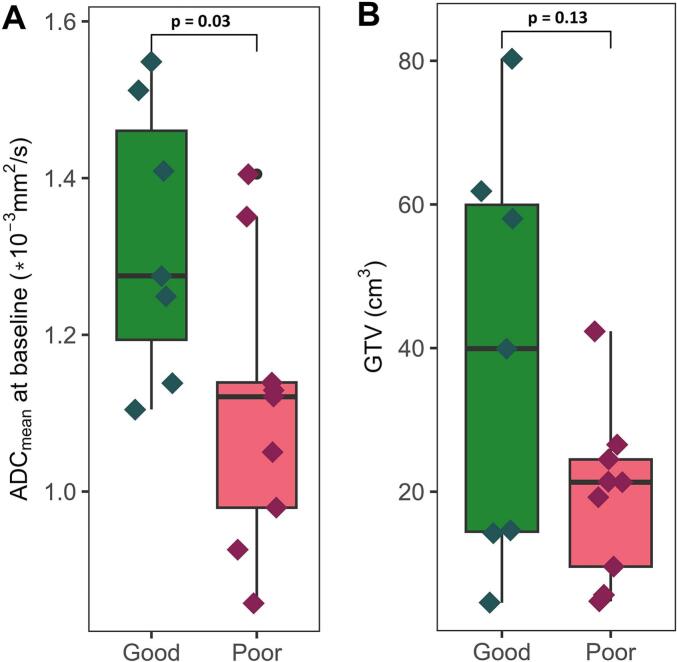
Baseline measurements of mean tumour apparent diffusion coefficient (ADC_mean_) (A) and volume (B) grouped into good (green) and poor (red) responders. Statistical difference tested by two-tailed *t*-test. The GTV is delineated on the high b-value DWI with the corresponding T2-weighted image as reference. (For interpretation of the references to colour in this figure legend, the reader is referred to the web version of this article.)

At baseline (Fig. 2), tumour ADC_mean_ was significantly higher in the good responding group compared to the poor responding group with median (range) ADC_good_ = 1.3 (1.1–1.5) ∙ 10^-3^ mm^2^/s and ADC_poor_ = 1.1 (0.9–1.4) ∙ 10^-3^ mm^2^/s (p = 0.03), respectively. There was no significant difference in initial tumour volume with median (range) GTV_good_ = 39.9 (4.6–80.3) cm^3^ and GTV_poor_ = 21.3 (4.7–42.3) cm^3^ (p = 0.13).

**Fig. 3:**
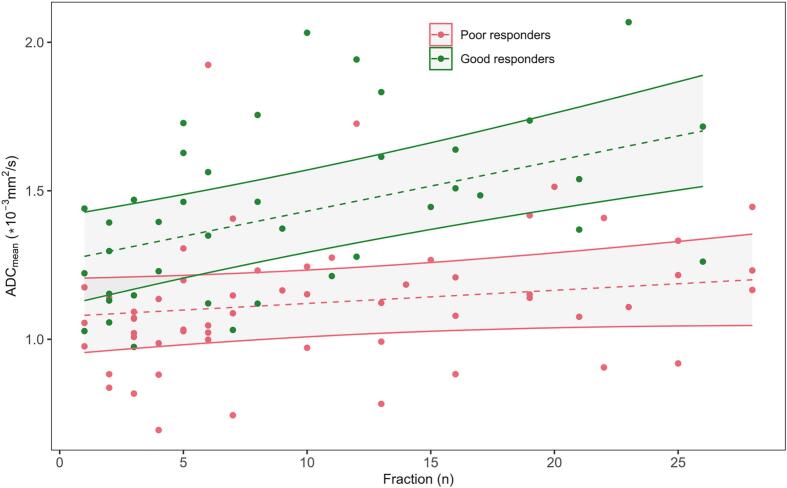
Mean tumour apparent diffusion coefficient (ADC_mean_) as a function of fraction, including all datapoints divided in good (green) and poor (red) responders. The fixed effect from the linear mixed-effects model is shown as dotted lines, including the respective 95% prediction band. (For interpretation of the references to colour in this figure legend, the reader is referred to the web version of this article.)

**Fig. 4:**
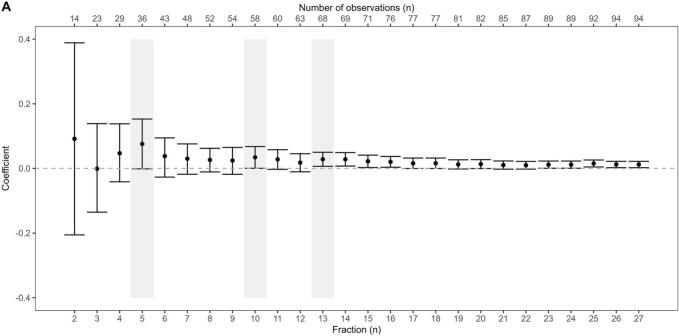

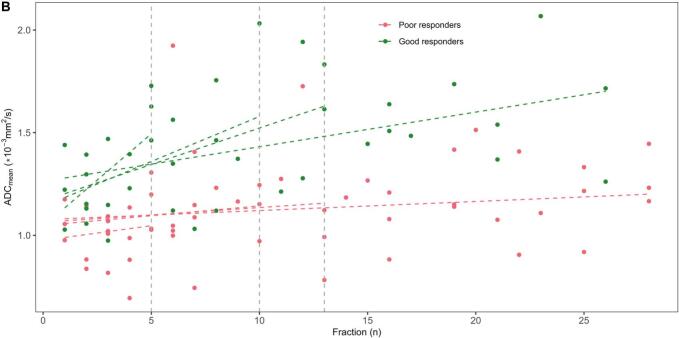
Cut-off analysis (A) and corresponding models (B). The estimated interaction term coefficient (β_3_) is shown with the confidence interval in the linear mixed-effects model. The lower x-axis shows the fraction cut-off while the respective number of observations included is shown in the upper x-axis. The shaded area highlights the chosen timepoints for further analysis. The corresponding model plot shows mean tumour apparent diffusion coefficient (ADC_mean_) as a function of fraction, divided in good (green) and poor (red) responders. The fixed effects from the linear mixed-effects models are given as dotted lines, with different slopes depending on fraction cut-off for model inclusion (from A, vertical lines). (For interpretation of the references to colour in this figure legend, the reader is referred to the web version of this article.)

The linear mixed-effects model using the total dataset revealed a significant impact on ADC_mean_ change per fraction by response (β_3_ = 0.012, CI [0.003, 0.022], p = 0.01) (Fig. 3). Investigating the ideal timepoint for discrimination of response by building the model with an increasing number of fractions resulted in no clear optimum but was close to significance at five (β_3_ = 0.076 (CI [-0.001,0.153], p = 0.06)) and ten fractions (β_3_ = 0.034 (CI [0.000,0.068], p = 0.05)), and then stabilised after around thirteen fractions (Fig. 4A). The same analysis applied to the linear-mixed effect of the volume showed a gradual stabilisation with the interaction term reaching significance after approximately three weeks (Supplementary Fig. S2). The ERI calculation resulted in an AUC of 0.67.

**Fig. 5:**
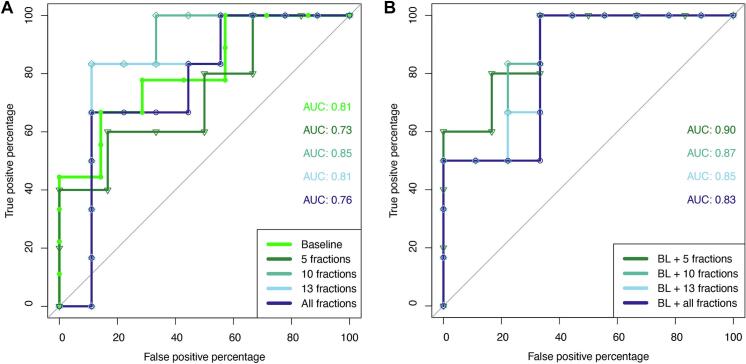
The receiver operating characteristic (ROC) curves for mean tumour apparent diffusion coefficient (ADC_mean_) at baseline and patient specific linear regression slopes at given cut-offs (A) and when combining baseline and slope at cut-offs (B).

Proceeding with patient specific linear regression models based on the cut-offs from the coefficient analysis, a more prominent change was found at earlier timepoints compared to the inclusion of all fractions. The largest increase was seen when including only five fractions, resulting in a slope of 0.090 and 0.014 per fraction for good and poor responders, respectively (Fig. 4B). The associated ROC curves provided the highest AUC value when including acquisitions from the first ten fractions (Fig. 5A, AUC_5 fr_ = 0.73, AUC_10 fr_ = 0.85, AUC_13 fr_ = 0.81, AUC_All fr_ = 0.76). When only the five first fractions were included, four patients were excluded due to zero or singular measurements from the first week (Supplementary Table S2). Combining baseline and slope resulted in small improvements in AUCs, with the highest score from the AUC combining baseline and the slope after five fractions (Fig. 5B, AUC_BL +5 fr_ = 0.90, AUC_BL + 10 fr_ = 0.87, AUC_BL + 13 fr_ = 0.85, AUC_BL + All fr_ = 0.83).

## 4 Discussion

In our study, sequential DW image acquisition revealed a distinction in ADC change among patients with good and poor response to CRT, an important prognostic factor in disease free survival in LARC. The difference in slope was most prominent at early timepoints, where the combination of baseline and sequential ADC assessment resulted in an AUC of 0.90 after only one week of treatment.

Decreased cellularity measured by an increase in ADC from baseline to post-CRT has been associated with good tumour response [15,19]. Our linear mixed-effects model revealed a significant interaction between fraction and response, implying that the level of ADC change during CRT is dependent on response (Fig. 3, Fig. 4). Early changes in ADC have previously been shown to be more reliable than early changes in volume, in agreement with our observation of a later ideal time-point for volume change evaluation (Supplementary Fig. S2) [18,20].

The indication of an ideal ADC-change evaluation at an early timepoint during CRT may also come from the potentially non-linear ADC trend over time (Supplementary Fig. S1). Significant differences in relative change in ADC between good and poor responders have previously been seen after two or three weeks [[17], [18], [19],^22^], supported by Sun et al. who found the largest difference in mean percentage of change already at the end of the first week [20]. The predictive value of our combined model was comparable to the previously reported AUC of 0.93 for the ERI biomarker after twelve fractions of CRT (BED = 25.5 Gy) [29], though with the possible benefit of stratification information already after five fractions (BED = 10.6 Gy). The large discrepancy between this AUC value for ERI reported by Cusumanu et al. and our findings of an AUC of 0.67 should be interpreted with caution. Notably, our study was not specifically designed to include the ERI calculation, with a classification of good and poor responders rather than pCR and non-pCR as well as varying input timepoints and volumes defined on DW images.

Low pre-CRT mean tumour ADC has previously been associated with a more aggressive tumour behaviour and higher local recurrence rate [19,[30], [31], [32]]. Still, studies on baseline data are inconsistent with respect to correlation to CRT response [15,31,33]. We found significantly higher baseline ADC in good responders compared to poor responders (Fig. 2A), in line with, and at the same level as, the findings of Palmisano et al. [31]. The baseline AUC of 0.81 might question whether sequential analysis from CRT is needed at all, though the increase in AUC by combining baseline and early CRT changes indicates a more reliable stratification using the coupled information. Noteworthy, the AUC of 0.90 using baseline and only the five first fractions can be relevant for hospitals lacking a combined MRI and radiotherapy system, as most of the patients (7/11) had only two acquisitions from this first week hence providing a logistically realistic scheduling option also on a diagnostic scanner (Supplementary Table S2). This early point of response assessment can enable further personalisation of treatment, such as indicating actions to be taken to increase response rates for watch-and-wait strategies.

Varying results in previous studies may be due to differences in image acquisition and ADC calculation among treatment facilities [34]. In a recent test–retest study by Eijkelenkamp et al. a relative repeatability coefficient of 17 % was determined for ADC on an MR-Linac system [35]. Previous feasibility studies using online or shuttle-based off-line approaches for ADC analysis, all capture the increase in ADC caused by CRT, but do not demonstrate a clear difference in trend between good and poor responders [[36], [37], [38]]. The limited sample size in these studies, ranging from three to eight patients, preclude strong conclusions about the possible relationship. Our use of mixed-effects modelling for early response account for intra- and inter-patient variability and unbalanced measurements, where our findings support that the change in ADC is more dominant than the repeatability uncertainty.

Previous studies also differ in VOI definition and response classification. Whether or not the entire lesion is evaluated might explain why some studies reported a detectable early response to CRT, while others did not [[19], [20], [21]]. The results from Kim et al. did not present this early-change difference, even when grouping the patient responders by three different criteria, though using a single slice rather than VOI for ADC analysis [21]. We chose to include the complete tumour statistics summarised as a mean value to ensure VOI-delineation reproducibility. Alternatively, only focusing on a hypointense region of the tumour could improve reproducibility in ADC, as proposed by Bisgaard et al. [39]. In regard to response classification, a study by Palmisano found the ADC values in near-CR to be in close relation to the non-responders, which may blur the difference in ADC trends [31]. We chose to define good responders as not only pCR but Dworak 3 or 4, as also patients with intermediate regression can improve their long-term outcome and still benefit from the prescribed treatment [40]. This broader definition of patients in the favourable group likely explains the more even distribution between good and poor responders in our cohort compared to studies grouping by pCR [4,5].

A limitation of the current study is the lack of investigation of the inter- and intra-observer variability in ADC tumour delineation, though using the same, well-experienced reader possibly contributed to consistent VOI delineations. Further, the analysis is simplified by the assumption of a linear change over time, which is probably a less accurate approximation reaching the end of the CRT course (Supplementary Fig. S1). When the patient inclusion started in 2018, the MRI-guided linear accelerator was recently installed at the hospital and the ideal utility and logistics were not yet established, seen by the varying acquisition timepoints. The change of image protocol (EPI3b) affected one patient in each response group. However, in the context of another publication, no significant difference in terms of ADC fitting were found between images from the two protocols [41]. The tumour shrinkage during CRT resulted in a higher number of datapoints in the beginning of the treatment, though visual inspection revealed the same bias in both groups. Adding more predictors could increase model accuracy. Due to the relatively small sample size, this next step was not considered appropriate for our study. Lastly, the analysis was restricted to volumes down to 4.6 cm^3^, as smaller volumes were deemed inappropriate for accurate delineation, thus this differentiation method does not apply to even smaller tumour volumes.

In conclusion, combining baseline and sequential ADC_mean_ information may provide a stratifying response assessment already after the first week of CRT. Though promising, these results need to be confirmed by larger multicentre studies.

## Declaration of competing interest

The authors declare the following financial interests/personal relationships which may be considered as potential competing interests: D. Thorwarth is Editor-in-Chief. S. Pilskog and K.R. Redalen are members of the Editorial Board. S. Pilskog reports an investigator-initiated grant from Varian Medical System inc., a Siemens Healthineers Company (project entitled: Improving current treatments of locally advanced rectal cancer through online adaptive radiotherapy with intensified chemotherapy). J.A. Hundvin is PhD-student on that grant. S. Pilskog receives funding from the Norwegian Cancer Society (grant number: 223048-2021). Tübingen reports financial and non-financial support by Elekta AB, Philips, Dr. Sennewald Medizintechnik, Therapanacea, Brainlab, PTW Freiburg in the context of institutional collaborations. C. Gani reports honoraria and travel sponsoring from Elekta.
