# Supplementary material for: Early response prediction during radiotherapy in rectal cancer using sequential diffusion-weighted imaging at a magnetic resonance image-guided linear accelerator
Source: Phys Imaging Radiat Oncol. 2025 Oct 8;36:100846. doi: 10.1016/j.phro.2025.100846 (PMC12550183; doi:10.1016/j.phro.2025.100846)
Supplement: Supplementary Data 1 [file mmc1.docx]

Supplementary material

Table S1: Parameter details for both diffusion-weighted sequences.

| **DWI:** | EPI4b (Patient 1-13^a^ and 16) | EPI3b (Patient 14-15) |
| --- | --- | --- |
| Sequence | SS-SE-EPI^b^ | SS-SE-EPI |
| Water-fat-shift (pix)/bandwidth (Hz) | 10.645/20.4 | 10.645/20.4 |
| EPI factor | 63 | 63 |
| Fold-over direction | RL | RL |
| Fat saturation | SPAIR | SPAIR |
| Number of signals averaged (NSA) | 1 | 1 |
| b-values (No. of excitations (NEX)) | 0 (2), 200 (3), 500 (4), 800 (6) | 0 (2), 150 (3), 500 (9) |
| TE/TR (ms) | 77/8097 | 68/7580 |
| Parallel imaging (SENSE factor) | 2.3 | 2.3 |
| Acquisition voxel size (mm^2^) | 3x3 | 3x3 |
| Field of view (mm^3^) | 430x430x192 | 430x430x192 |
| Slice thickness (mm) | 4.8 | 4.8 |
| Slice gap (mm) | 0 | 0 |
| Scan duration | 5:40 min | 4:56min |

^a^ Patient 6’s 7^th^ fraction was acquired with b-values 50, 500 and 800. In this case, the ADC was calculated using the b-values 50 and 500.
^b^ Single-shot spin-echo echo-planar-imaging

Table S2: Acquisition overview per patient (P) showing stage and MRF/EMVI status, image data included in the analysis (x) and acquisitions excluded due to artefacts (a), too small tumour size for accurate delineation (s), image only acquired with rectal filling (f) or deemed redundant (already a sufficient number of images included) (r). Good responders are marked with bold green.

|  | Fraction | | | | | | | | | | | | | | | | | | | | | | | | | | | |  |  |  |
| --- | --- | --- | --- | --- | --- | --- | --- | --- | --- | --- | --- | --- | --- | --- | --- | --- | --- | --- | --- | --- | --- | --- | --- | --- | --- | --- | --- | --- | --- | --- | --- |
| P | 1 | 2 | 3 | 4 | 5 | 6 | 7 | 8 | 9 | 10 | 11 | 12 | 13 | 14 | 15 | 16 | 17 | 18 | 19 | 20 | 21 | 22 | 23 | 24 | 25 | 26 | 27 | 28 | Stage | MRF | EMVI |
| **1** |  | **x** |  |  | **x** |  |  | **x** |  | **x** |  | **x** |  |  |  | **x** |  |  | **x** |  |  |  | **x** |  |  | **s** |  | **s** | **T3N2** | **-** | **-** |
| 2 |  | x |  | x |  |  | x |  |  | x |  |  |  | x |  | x |  |  | x |  |  | x |  |  | x |  |  | x | T3N1 | + | - |
| **3** |  | **x** |  |  | **x** |  |  |  | **x** |  |  | **x** |  |  | **x** |  |  | **s** |  |  | **s** |  |  | **s** |  |  | **s** |  | **T3N1** | **-** | **-** |
| 4 |  |  | x |  | x |  | x |  |  | s | s |  | s |  |  | s |  |  | s |  |  | s |  |  | s |  | s |  | T2N1 | - | - |
| **5** |  |  | **x** |  |  | **x** |  |  | **s** |  |  | **s** |  |  | **s** |  |  | **s** |  |  | **s** |  |  | **s** |  |  | **s** |  | **T3N2** | **-** | **-** |
| 6 |  |  | x |  |  |  | x |  |  | x |  |  | x |  |  | x |  |  | x |  | x |  |  |  | x |  |  |  | T3N0 | - | + |
| 7 |  |  |  |  |  | x |  |  | x |  |  | x |  |  |  |  |  |  |  | x |  |  |  |  | x |  |  | x | T3N1 | + | - |
| 8 |  |  | x |  |  |  | x |  |  |  |  |  | x |  |  | x |  |  |  |  |  | x |  |  |  | s |  |  | T3N2 | + | - |
| 9 | x | x | x | x | x |  |  | s |  |  |  |  | s |  |  |  |  | s | s |  |  |  |  | s |  |  |  | s | T3N1 | - | + |
| **10** | **x** | **x** | **x** | **x** |  |  |  | **x** |  |  |  |  | **x** |  |  | **s** |  |  |  |  | **s** |  |  |  |  | **s** |  |  | **T4N2** | **+** | **+** |
| 11 |  |  | a | x | x | x |  | f |  |  |  |  | x |  |  |  |  |  |  | f |  |  | x | f |  |  |  | x | T3N1 | - | - |
| **12** | **x** | **x** | **x** | **x** | **x** | **x** |  |  |  |  |  |  | **x** |  |  |  | **x** |  |  |  | **x** |  |  |  |  | **x** |  |  | **T3N1** | **-** | **+** |
| 13 | x | x | x | x | x | x |  |  |  | x |  |  |  |  | x |  |  |  | x |  |  |  |  | s |  |  | s |  | T3N0 | + | - |
| 14 | x |  | x |  |  | x |  | x |  |  | x |  | s |  |  | s |  | s |  | s |  |  | s | s |  | s |  | s | T3N2 | - | - |
| **15** | **x** |  | **x** |  |  | **x** | **x** | **x** |  |  | **x** | **r** | **r** |  |  | **x** | **r** | **r** |  |  | **x** | **r** |  | **r** |  | **x** | **r** | **r** | **T4N2** | **+** | **-** |

*MRF: Mesorectal fasia; EMVI: Extramural venous invasion*


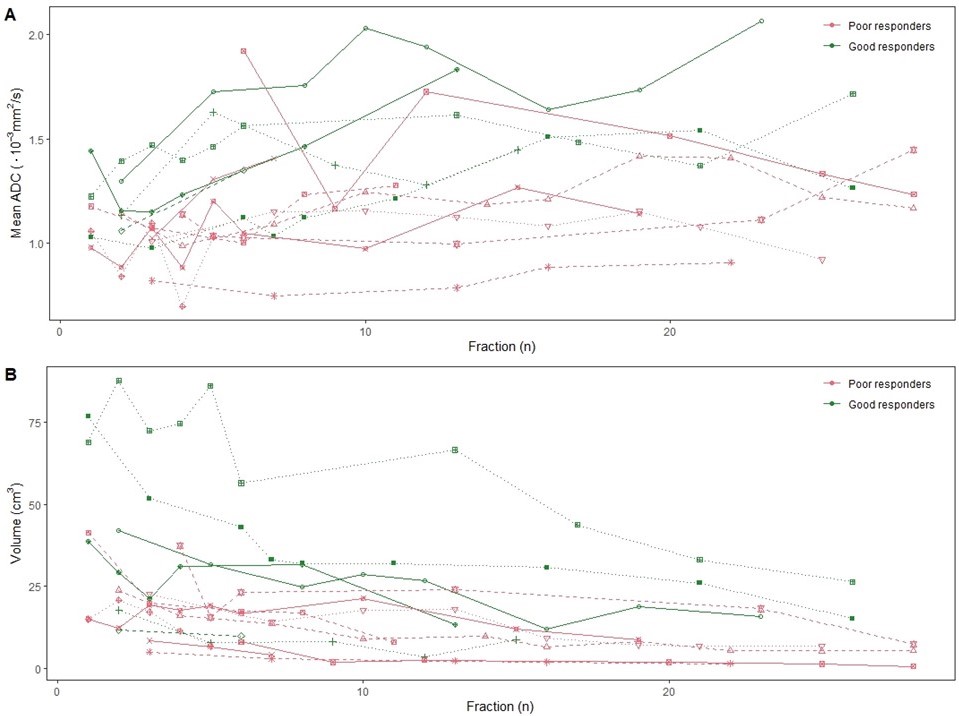


Figure S1: Individual trends in tumour mean ADC (A) and volume (B), labelled in good (green) and poor (red) responders.


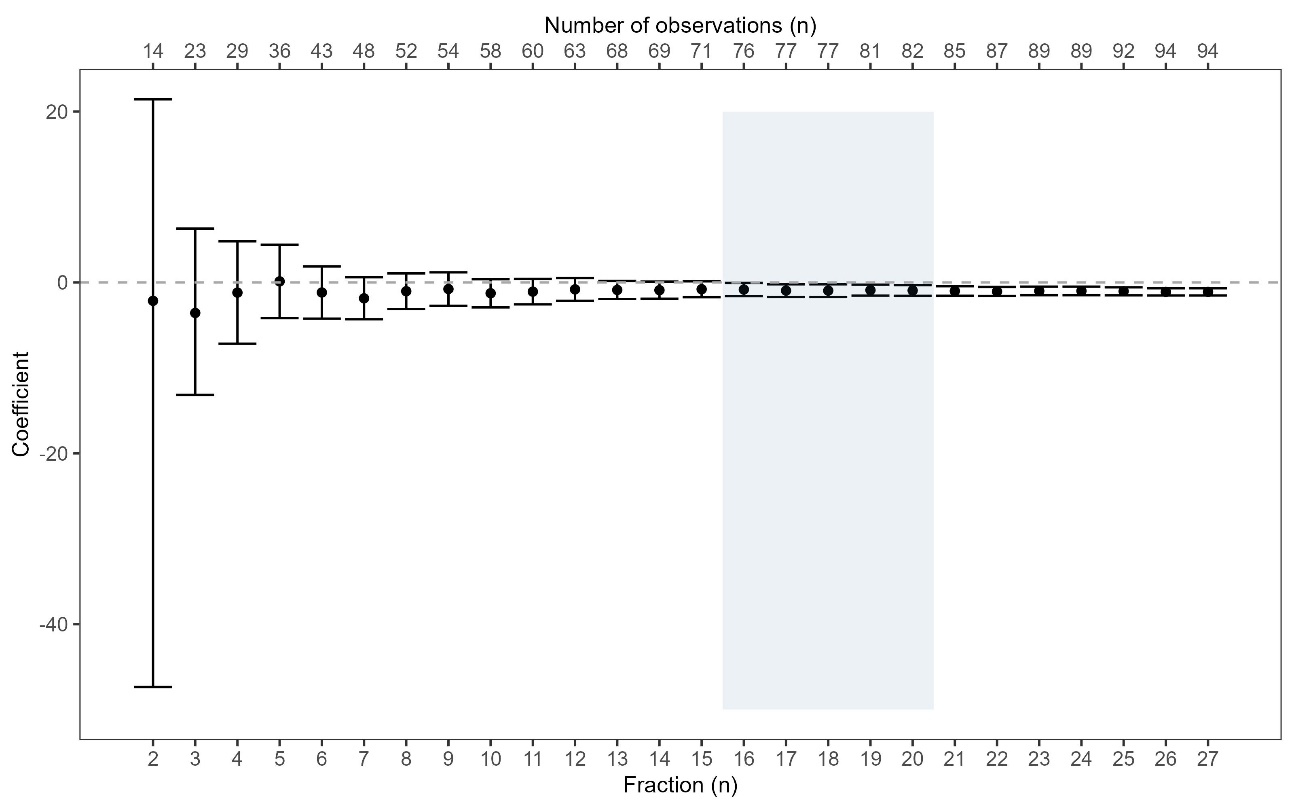


Figure S2: Estimation of the interaction term coefficient (β_3_) with confidence interval in the linear mixed-effects model using volume as dependent variable. The lower x-axis shows the fraction cut-off and the upper x-axis the respective number of observations included. The shaded area highlights the area where the interaction term stabilises as significant. NB: Different y-axis scale than Figure 3 for ADC.
